# Identifying key biomarkers and therapeutic candidates for post-COVID-19 depression through integrated omics and bioinformatics approaches

**DOI:** 10.1515/tnsci-2022-0360

**Published:** 2024-11-23

**Authors:** Yi Zhou, Chunhua Yang, Jing Zhou, Qiyao Zhang, Xingling Sui, Hongyu Dong, Haidong Zhang, Yue Wang

**Affiliations:** Department of Emergency, Shandong Provincial Hospital Affiliated to Shandong First Medical University, Jinan, Shandong, 250021, China; School of Clinical and Basic Medical Sciences, Shandong First Medical University & Shandong Academy of Medical Sciences, Jinan, 250117, China; Medical Experimental Center, Jinan Maternity and Child Care Hospital Affiliated to Shandong First Medical University, Jinan, 250000, China

**Keywords:** COVID-19, major depressive disorder, neuroinflammation, neuroimmune response, transcriptional and posttranscriptional regulation

## Abstract

**Introduction:**

Depression, the leading cause of disability worldwide, is known to be exacerbated by severe acute respiratory syndrome coronavirus 2 infection, worsening coronavirus disease 2019 (COVID-19) outcomes. However, the mechanisms and treatments for this comorbidity are not well understood.

**Methods:**

This study utilized Gene Expression Omnibus datasets for COVID-19 and depression, combined with protein–protein interaction networks, to identify key genes. Gene ontology and Kyoto Encyclopedia of Genes and Genomes analyses were performed to understand gene functions. The CIBERSORT algorithm and NetworkAnalyst were used to examine the relationship of immune cell infiltration with gene expression and to predict transcription factors (TFs) and microRNAs (miRNAs) interactions. The Connectivity Map database was used to predict drug interactions with these genes.

**Results:**

*TRUB1*, *PLEKHA7*, and *FABP6* were identified as key genes enriched in pathways related to immune cell function and signaling. Seven TFs and nineteen miRNAs were found to interact with these genes. Nineteen drugs, including atorvastatin and paroxetine, were predicted to be significantly associated with these genes and potential therapeutic agents for COVID-19 and depression.

**Conclusions:**

This research provides new insights into the molecular mechanisms of post-COVID-19 depression and suggests potential therapeutic strategies, marking a step forward in understanding and treating this complex comorbidity.

## 1 Introduction

Depression, a leading cause of global disability, has experienced a significant increase in prevalence in the wake of the coronavirus disease 2019 (COVID-19) pandemic [1]. The swift spread of the virus, societal dread, and physical toll of the infection synergistically increased the incidence of depressive symptoms among COVID-19 patients, with prevalence estimates ranging from 11 to 28%. Strikingly, a subset of 3–12% of these individuals endures severe depressive disorders [2]. The interplay between severe acute respiratory syndrome coronavirus-2 (SARS-CoV-2) infection and depression is particularly concerning, as it has been demonstrated to markedly worsen the prognosis of patients with COVID-19 [3].

Previous research has indicated that individuals with preexisting respiratory conditions are more susceptible to concurrent depression, highlighting the intricate link between respiratory health and mental well-being [4]. The psychological aftermath of SARS-CoV-2 infection, characterized by persistent inflammation and the body’s immune response, is believed to be a key driver of post-COVID-19 depression [5,6]. The invasion of host cells via the angiotensin-converting enzyme (ACE2) receptor triggers a cascade of events, including the activation of lymphocytes and the subsequent release of proinflammatory cytokines and chemokines [7–9]. This immune response, coupled with the stress-induced activation of immune cells, sets the stage for depressive symptoms [5,10].

Interestingly, a decrease in ACE2 levels has been observed in individuals with depression, suggesting a shared pathophysiological mechanism between depression and the inflammatory response elicited by COVID-19 [11,12]. The presence of ACE2 on the cell surface and the production of inflammatory cytokines are thus considered pivotal in the co-development of these conditions.

While existing research has delved into the origins of COVID-19-associated depression, the precise molecular mechanisms involved remain largely elusive [13]. To address this gap, our study leverages publicly available gene expression and clinical data from individuals with COVID-19 and depression, employing sophisticated bioinformatics tools to identify key genes and explore their underlying mechanisms.

We constructed a network of transcription factors (TFs) and microRNAs (miRNAs) that interact with these pivotal genes, revealing a complex regulatory system. Furthermore, our analysis identified a series of molecular drugs that may serve as potential treatments for both COVID-19 and depression. By uncovering these core biomarkers and their associated mechanisms, this research paves the way for a more targeted and effective therapeutic approach to managing the dual burden of COVID-19 and depression.

## 2 Materials and methods

### 2.1 Data acquisition, processing, and identification of differentially expressed genes (DEGs) common to COVID-19/depression

We sourced raw expression profile data from four datasets: two focused on COVID-19 (GSE164805 and GSE171110) and two focused on depression (GSE201332 and GSE135524). All datasets included samples from peripheral blood, with detailed sample compositions, including both healthy control samples and patient samples, as summarized in Table 1. To mitigate the impact of batch effects across the integrated datasets, we strategically employed the “combat” algorithm. This method, which is part of the robust sva package in R, is designed to adjust for batch effects, thereby ensuring the comparability of gene expression data across different experimental conditions. We subsequently utilized the “Limma” package, a widely recognized tool in bioinformatics, for the identification of DEGs. This package facilitated a comparative analysis between the COVID-19 and moderate depression groups and the healthy control and mild depression groups. We applied a stringent threshold of |log2 (fold-change)| ≥0.58 and *P* < 0.05 to ensure that only the most significantly altered genes were considered, enhancing the reliability of our findings. To further refine our data, the “normalizeBetweenArrays” method was employed to standardize the expression data across all samples. This normalization step is crucial, as it adjusts for technical variability, allowing for more accurate comparisons and reducing the risk of false positives in DEG identification. Following this, the “lmFit” function was applied to perform a nonlinear least squares analysis of the data. This statistical approach is valuable for modeling the relationship between gene expression and conditions, providing a robust framework for the analysis of complex gene expression data. Finally, the “eBayes” function was implemented to adjust the variance estimates, which is essential for improving the accuracy of statistical inference. This function employs Bayesian methods to moderate the standard errors, enhancing the reliability of the statistical significance testing. By intersecting the upregulated and downregulated DEGs across the three conditions, we identified genes commonly associated with COVID-19 and depression.

**Table 1:** Information from the peripheral blood of all four datasets, including two COVID-19 datasets, GSE164805 and GSE171110, and two depression datasets, GSE201332 and GSE135524

	GSE135524		GSE201332		GSE171110		GSE164805	
	Mild depression	Moderate depression	Control	MDD	Control	COVID-19	Control	COVID-19
Sample number	*n* = 33	*n* = 55	*n* = 20	*n* = 20	*n* = 10	*n* = 44	*n* = 5	*n* = 10
Sample type	Peripheral blood		Peripheral blood		Peripheral blood		Peripheral blood	
Experimental technique	RNA sequencing		Microarray		RNA sequencing		Microarray	
Patient population	American		Chinese		France		Chinese	

### 2.2 Assessment of the diagnostic efficacy of key genes

To evaluate the diagnostic potential of the identified DEGs for COVID-19 and depression, we constructed receiver operating characteristic (ROC) curves and calculated the area under the curve (AUC) via the “pROC” R package.

### 2.3 Functional enrichment analysis and protein–protein interaction (PPI) network analysis were used to identify hub genes

We conducted gene ontology (GO) and Kyoto Encyclopedia of Genes and Genomes (KEGG) pathway enrichment analyses of the key DEGs via the “clusterProfiler” package. The “ggplot2” package facilitated the visualization of these enrichment results. We subsequently utilized the STRING database within the NetworkAnalyst platform (https://www.networkanalyst.ca) to perform PPI network analysis, setting a medium confidence score cutoff at greater than 400. The resulting network was visualized via Cytoscape software, and genes with a high degree of centrality and betweenness centrality, indicative of their roles as pivotal signaling nodes, were selected as hub genes.

### 2.4 Functional enrichment analysis of the hub genes

COVID-19 samples were stratified based on median expression levels of the identified hub genes. We then isolated the corresponding DEGs via the “Limma” package, applying the same thresholds as previously mentioned. The “clusterProfiler” package was again employed to conduct GO and KEGG enrichment analyses for these DEGs in relation to the hub genes.

### 2.5 Correlation analysis between immune cell infiltration and hub gene expression

The CIBERSORT algorithm was used to investigate the relationship between the infiltration levels of immune cells and the expression of hub genes. Pearson correlation analysis was used to quantify the associations between hub gene expression and immune checkpoint levels, with a *P*-value <0.05 considered indicative of a significant correlation.

### 2.6 TF–miRNA–hub gene coregulatory network construction

NetworkAnalyst was used to predict potential TFs and miRNAs that may regulate the hub genes via linked databases such as JASPAR, TarBase, and RegNetwork. The resulting TF–miRNA–gene interaction network was visualized via Cytoscape software.

### 2.7 Candidate drug identification

Utilizing the CMAP online database (https://clue.io/), we analyzed the potential interactions between the hub genes and associated drugs. We filtered for candidate therapeutic compounds with a connective score absolute value exceeding 90, which may have significant implications for treatment.

## 3 Results

### 3.1 Identification and analysis of common DEGs

**Figure 1:**
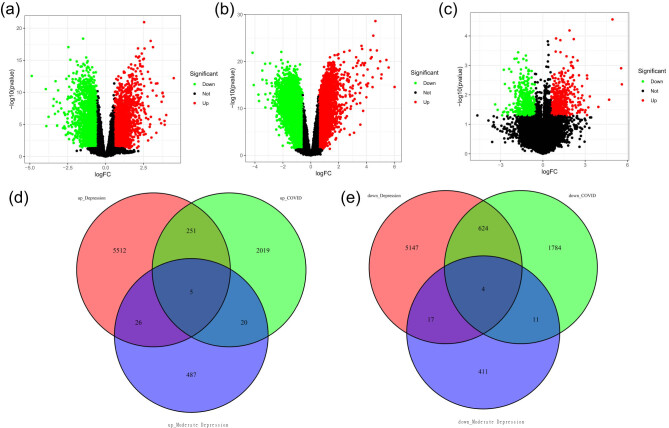
Identifying DEGs common to both COVID-19 and depression. (a) Volcano plot of the DEGs in COVID-19 patients from the GSE164805 and GSE171110 datasets. (b) Volcano plot of the DEGs associated with depression from the GSE201332 dataset. (c) Volcano plot of the DEGs associated with depression from the GSE135524 dataset, which included mild and moderate depression cohorts. (d) Intersection of upregulated DEGs in COVID-19 patients and upregulated DEGs in depression and moderate depression patients. (e) Intersection of downregulated DEGs in COVID-19 patients, with downregulated DEGs associated with depression and mild depression.

Our initial analysis yielded 4,718 DEGs from the comparison between the COVID-19 and control groups, comprising 2,295 upregulated and 2,423 downregulated genes (Figure 1a). In the context of depression, the GSE201332 and GSE135524 datasets revealed a total of 11,586 and 981 DEGs, respectively, with 5,794 and 538 upregulated genes and 5,792 and 443 downregulated genes (Figure 1b and c). By intersecting the DEGs from the three conditions, we identified a set of five positively correlated common DEGs, namely, *COL10A1*, *FABP6*, *INSM2*, *SPSB4*, and *SLC10A2*, and four negatively correlated common DEGs, namely, *WDR89*, *CCDC102B*, *PLEKHA7*, and *TRUB1* (Figure 1d and e).

### 3.2 Validation of the expression and diagnostic utility of key genes

**Figure 2:**
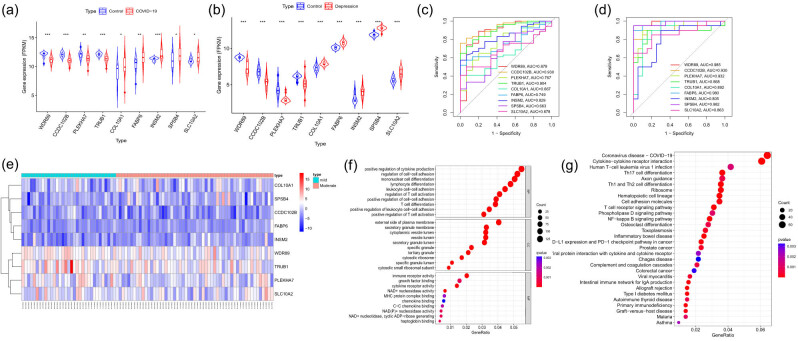
Elaborating the expression and diagnostic efficacy of key genes in COVID-19/depression prevention and functional enrichment analysis. (a) Expression levels of the key DEGs in COVID-19. (b) Expression levels of the key DEGs associated with depression. (c) AUC values of the ROC curves of these genes in patients with COVID-19. (d) AUC values of the ROC curves of these genes in patients with depression. (e) A heatmap illustrating the expression distributions of key genes in the mild and moderate depression cohorts. (f) GO enrichment of key genes. (g) KEGG enrichment of key genes.

The expression patterns of the common DEGs associated with COVID-19 and depression are shown in Figure 2a and b, with the corresponding ROC curves depicted in Figure 2c and d. Notably, *COL10A1*, *FABP6*, *INSM2*, *SPSB4*, and *SLC10A2* presented increased expression in both conditions relative to controls, whereas *WDR89*, *CCDC102B*, *PLEKHA7*, and *TRUB1* presented decreased expression within the disease groups. The AUC values for these genes in depression patients surpassed 0.8, and those in COVID-19 patients exceeded 0.7, with the exception of *COL10A1*, *SPSB4*, and *SLC10A2*, whose AUC values were less than 0.7. The heatmap in Figure 2e provides a visual representation of the expression distribution of these key genes across the mild and moderate depression cohorts. Subsequent GO enrichment analysis of the common DEGs revealed significant enrichment in processes such as T-cell differentiation, lymphocyte differentiation, and positive regulation of cytokine production (Figure 2f). KEGG pathway analysis further revealed that these key genes are involved in pathways such as Th1 and Th2 cell differentiation, Th17 cell differentiation, and the T-cell receptor signaling pathway (Figure 2g). These results showed that the immune response played a significant mediating role in the pathogenesis of both pandemics.

### 3.3 Construction and analysis of the PPI network to identify hub genes and their functional enrichment

**Figure 3:**
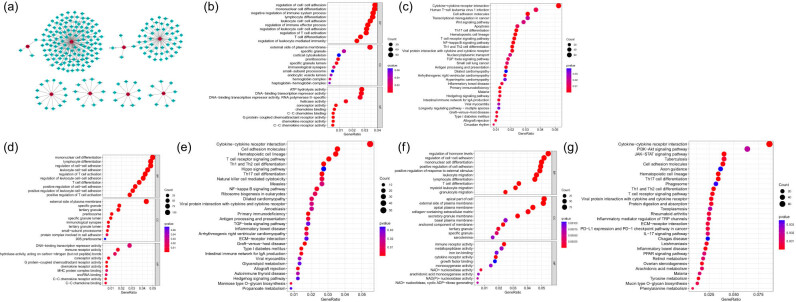
PPI network construction to identify hub genes and functional enrichment analyses for hub genes. (a) The NetworkAnalyst platform was used to construct a PPI network for key genes, and three hub genes, *TRUB1*, *PLEKHA7*, and *FABP6*, were screened. (b)–(g) GO and KEGG enrichment analyses of DEGs corresponding to *TRUB1* (b and c), *PLEKHA7* (d and e), and *FABP6* (f and g) were performed by separating them into high/low single-hub gene groups.

We constructed a PPI network for the identified genes via the NetworkAnalyst platform, which facilitated the extraction of three pivotal hub genes, *TRUB1*, *PLEKHA7*, and *FABP6*, on the basis of their degree and betweenness centrality rankings (Figure 3a). We subsequently conducted GO and KEGG enrichment analyses for DEGs corresponding to each hub gene, stratifying COVID-19 samples into high- and low-expression groups for each gene. *TRUB1* gene expression was predominantly associated with biological processes (BPs), such as mononuclear/lymphocyte/T-cell differentiation/adhesion, T-cell activation, immune effector, and systemic process regulation, as well as pathways involving cytokine–cytokine receptor interactions, Th17/Th1/Th2 cell differentiation, and Wnt/T-cell receptor/NF-κB/TGF-β signaling cascades (Figure 3b and c). *PLEKHA7* was associated with BPs and pathways similar to those of *TRUB1*, with a particular emphasis on mononuclear/lymphocyte/T-cell differentiation/adhesion and T-cell activation (Figure 3d and e). *FABP6* was linked to processes such as mononuclear/lymphocyte/T-cell differentiation, cell–cell adhesion, leukocyte/granulocyte migration, and pathways such as cytokine‒cytokine receptor interaction, PI3K-AKT/JAK-STAT/T-cell receptor/IL-17 signaling, Th17/Th1/Th2-cell differentiation, and phagosome function (Figure 3f and g). These results underscore the close associations of the hub genes with immune cell differentiation, migration, activation, immune process regulation, and multiple signaling pathways, as well as their interactions with cytokine–cytokine receptors.

### 3.4 Correlation analysis of hub genes with immune cell characteristics

**Figure 4:**
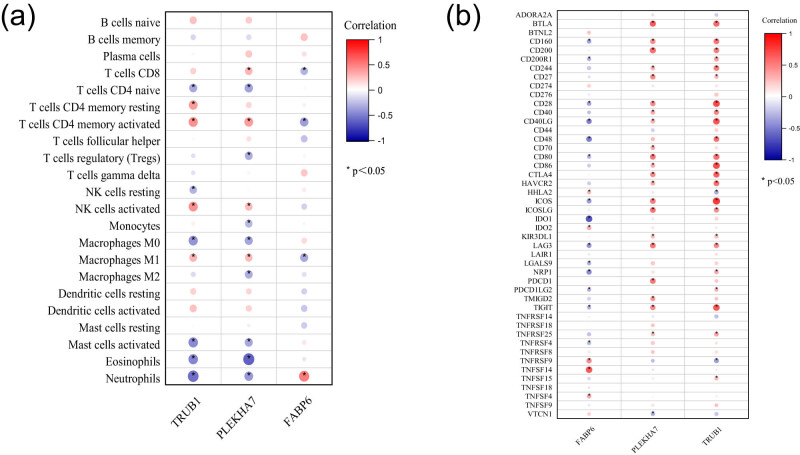
Immune characteristic correlation analyses of the three hub genes. (a) Correlation between hub gene expression and infiltrating immune cells. (b) Correlations between hub gene expression and immune checkpoint markers.

Given the enriched roles of the hub genes in immunological regulation and inflammatory responses, we employed the CIBERSORT algorithm to explore the relationships between the expression of these genes and the abundance of infiltrating immune cells. The correlation between hub gene expression and immune cell infiltration is depicted in Figure 4a. *TRUB1* was positively correlated with activated/resting memory CD4^+^ T cells, activated NK cells, and M1 macrophages but negatively correlated with naive CD4^+^ T cells, resting NK cells, M0 macrophages, activated mast cells, eosinophils, and neutrophils. *PLEKHA7* was positively associated with CD8^+^ T cells, activated memory CD4^+^ T cells, activated NK cells, and M1 macrophages but negatively correlated with naive CD4^+^ T cells, Tregs, monocytes, M0/M2 macrophages, activated mast cells, eosinophils, and neutrophils. In contrast, FABP6 was positively correlated with neutrophils and negatively correlated with CD8^+^ T cells, activated memory CD4^+^ T cells, and M1 macrophages, indicating an opposing pattern compared with the other two hub genes.

Moreover, the correlations between the hub genes and immune checkpoint markers indicated that *TRUB1* and *PLEKHA7* were positively related to these markers, whereas FABP6 had the opposite relationship (Figure 4b).

### 3.5 Establishment of the TFs–miRNAs–hub genes coregulation network

**Figure 5:**
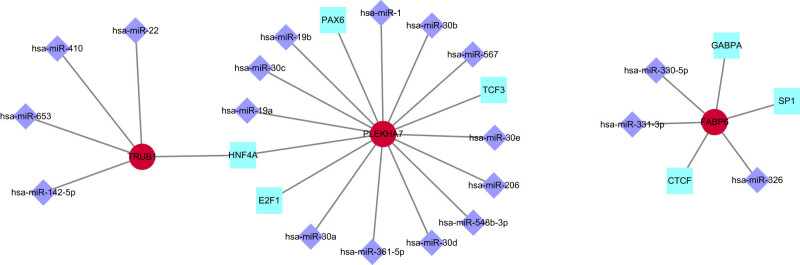
Construction of a TF–miRNA–hub gene coregulation network. A total of 7 TFs and 19 miRNAs were predicted to interact with the hub genes.

Gene expression is most commonly and significantly influenced by two primary regulatory mechanisms, transcriptional regulation mediated by TFs and posttranscriptional regulation mediated by miRNAs [14]. To elucidate the intricate regulatory mechanisms governing the expression of the identified hub genes, we utilized NetworkAnalyst to construct a comprehensive network of TFs and miRNAs. Our analysis revealed a complex interplay, with 7 TFs and 19 miRNAs interacting with the hub genes. Specifically, *TRUB1* was found to be under the regulatory influence of four miRNAs and one TF, which also had a concurrent effect on *PLEKHA7*. For *PLEKHA7*, 12 miRNAs and 4 TFs were identified as key regulators. *FABP6* was predicted to be influenced by two miRNAs and three TFs, as depicted in Figure 5.

### 3.6 Target drug prediction

**Figure 6:**
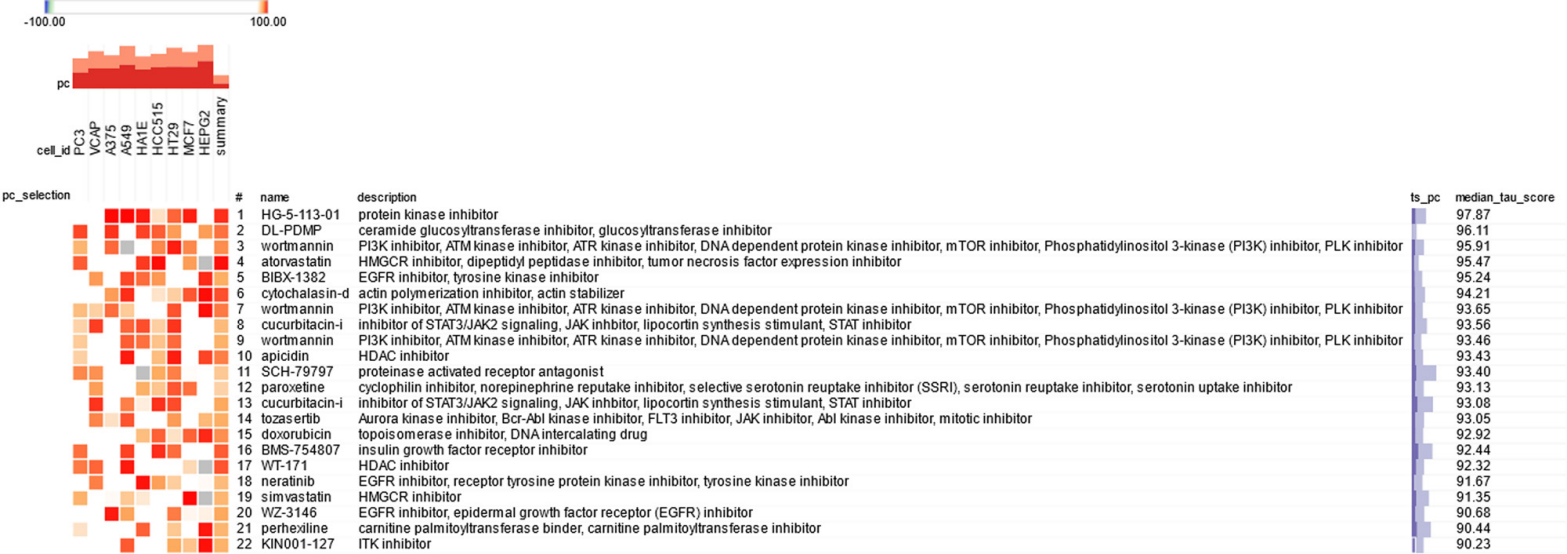
Candidate drugs were predicted. The use of CMAPs to predict 19 drugs closely correlated with COVID-19 and depression, with the limitation of a median tau score >90.

Employing the CMAP database, we identified a cohort of 19 molecular drugs that exhibited a strong correlation with the hub genes, as indicated by a median tau score exceeding 90. This selection included HG-5-113-01, DL-PDMP, wortmannin, atorvastatin, BIBX-1382, cytochalasin-d, cucurbitacin-i, apicidin, SCH-79797, paroxetine, tozasertib, doxorubicin, BMS-754807, WT-171, neratinib, simvastatin, WZ-3146, perhexiline, and KIN001-127, as illustrated in Figure 6. These drugs hold promise for the development of targeted molecular therapies aimed at combating both COVID-19 and depression, offering a new frontier in treatment strategies.

## 4 Discussion

A growing amount of evidence has highlighted a stark rise in the prevalence of mental health disorders, including depression, anxiety, and insomnia, amidst the ongoing COVID-19 pandemic [15]. Studies have consistently reported increased depressive and anxious symptoms in the early months of the pandemic compared with pre-pandemic levels [16]. Furthermore, research from Italy revealed a sustained increase in mental health symptoms, such as anxiety and depression, even into the second year following the pandemic’s onset [17].

Increasing research has suggested that alterations in brain structure and function, neural activity, and network connectivity may underpin the emergence of pandemic-related depression [12,18]. These findings are complemented by the role of the neuroimmune response, with factors such as neuroinflammation, compromised blood–brain barrier integrity, and the potential invasion of SARS-CoV-2 into the central nervous system contributing to the etiology of depression during the pandemic [19]. Elevated cytokine levels and disruptions in the gut microbiome have also been implicated, with the latter potentially driving excessive proinflammatory cytokine production and intestinal barrier damage, further exacerbating the risk of depression [20,21].

Despite these insights, the molecular mechanisms that link depression with COVID-19 remain to be fully elucidated. This study delves into these complexities, identifying key biomarkers, underlying functions, and pathways that are central to the interplay between the virus and mental health. By doing so, we have identified potential therapeutic targets, offering hope for the development of targeted interventions for COVID-19-induced depression.

In this study, the genes *TRUB1*, *PLEKHA7*, and *FABP6* emerged as central hubs potentially contributing to the pathogenesis of both depression and COVID-19. Our findings revealed that *TRUB1* and *PLEKHA7* expression was decreased, whereas FABP6 expression was increased in both the depression group and the COVID-19 patient group compared with the control group. Previous research by Zhang et al. revealed that low levels of *TRUB1*, a highly conserved pseudouridine synthase, are associated with an adverse prognosis in glioblastoma multiforme [22]. Disruptions in *PLEKHA7* expression have been shown to promote the delamination of basal progenitors, subsequently increasing their numbers and the neuronal population in the cortical plate [23]. Conversely, *FABP6*, which is upregulated in gliomas compared with control brain tissue, has been implicated in reduced invasion and angiogenesis upon *FABP6* knockdown [24]. Additionally, urinary intestinal fatty acid binding protein (urinary-FABP), recognized as a marker for gut injury, is correlated with increased mortality in pneumonia patients [25]. Heart fatty acid binding protein (heart-FABP) levels are elevated in COVID-19 patients, with higher levels correlating with disease severity [26]. Overall, this study highlights the significant roles of *TRUB1*, *PLEKHA7*, and *FABP6* in the development of depression associated with COVID-19.

Functional and pathway enrichment analyses are crucial for understanding the regulatory mechanisms of hub genes in COVID-19-related depression. Our investigation revealed the enrichment of processes such as mononuclear/lymphocyte/T-cell differentiation/adhesion, T-cell activation, immune regulation, and leukocyte/granulocyte migration. COVID-19 is known to cause a significant decline in CD4^+^ T cells, affect T-cell differentiation, and disrupt innate immune antigen presentation, as well as monocyte population imbalances [27,28]. T-cell activation is particularly important for neuroprotection and anti-inflammatory effects in major depressive disorder [29]. Furthermore, pathways involved in cytokine signaling, Th17/Th1/Th2 cell differentiation, and Wnt/T-cell receptor/NF-κB/TGF-β signaling are recognized as key regulatory pathways. The increased levels of inflammatory chemokines in ICU COVID-19 patients and the role of NF-κB in SARS-CoV-2 infection are well-documented [30–32], as is the involvement of TGF-β in pulmonary fibrosis caused by COVID-19 [33]. Moreover, the NF-κB pathway also plays a significant role in neuronal plasticity and neurogenesis related to depression, while the PI3K-Akt pathway is implicated in antidepressant activity and neuroprotection [34,35]. These biological functions and regulatory mechanisms are in accordance with those reported previously in patients with COVID-19 or depression; however, these mechanisms mediate depression in patients with COVID-19 infection.

This study also highlighted the link between hub genes, including T cells, NK cells, macrophages, eosinophils, neutrophils, Tregs, and immune cell activity. The associations between immune response biomarkers and the risk of depression in the context of COVID-19 remain an area of ongoing research.

Finally, in this study, we explored the transcriptional and posttranscriptional regulation of these key genes and screened potential targeted drugs. For the first time, we identified a series of TFs, miRNAs, and small molecule compounds, charting a course for future research endeavors. The identification of 19 molecular drugs as potential therapeutic candidates for COVID-19 and depression is important. These include DL-PDMP, which inhibits ganglioside synthesis [36], and wortmannin, which reduces ACE2 levels in mast cells [37]. Statins, such as atorvastatin and simvastatin, have been shown to alleviate depressive symptoms and provide protection against COVID-19 by modulating cytokine release and T-cell activity [38–40]. BIBX 1382, an EGFR inhibitor, and paroxetine, a serotonin reuptake inhibitor, are also among the potential therapeutic agents, along with doxorubicin, which has been repurposed as an anti-COVID-19 drug [41–43].

Certain limitations require attention. First, the dataset utilized in this article encompasses individuals from Chinese, French, and American patient populations, which introduces potential ethnic variations that could influence the reliability of our findings. Additionally, future research should aim to incorporate more nuanced data resources characterized by larger sample sizes and comprehensive clinical details. Furthermore, while this study is rooted primarily in computational biology, experimental validation and clinical confirmation studies must be conducted. Finally, it is imperative to elucidate the underlying mechanisms, including molecular regulation, pathway mediation, and target‒drug interactions, in subsequent investigations.

## 5 Conclusion

In summary, this research highlights the roles of *TRUB1*, *PLEKHA7*, and *FABP6* as hub genes, the underlying pathways and TF–miRNA networks, and the potential impact of these genes on immune cell activity in the development of depression and COVID-19. The identification of candidate drugs and potential regulatory mechanisms provides a foundation for optimizing predictive and therapeutic strategies for these intertwined conditions.
